# Combination Treatment of Carboxyl Esterase 2-Overexpressing hTERT-Immortalized Human Adipose Stem Cells Enhances the Inhibition of Tumor Growth by Irinotecan in PC3, a Castration-Resistant Prostate Cancer Model

**DOI:** 10.3390/cimb47110902

**Published:** 2025-10-30

**Authors:** Jae Heon Kim, Miho Song, Jeongkun Lee, Sang Hun Lee, Yun Seob Song

**Affiliations:** 1 Department of Urology, Soonchunhyang University School of Medicine, Seoul 04401, Republic of Korea; piachkjh@hanmail.net (J.H.K.); miho@schmc.ac.kr (M.S.); 2 Program in Biomedical Sciences, Department of Biomedical Sciences, College of Medicine, Engineering Inha University, Incheon 22212, Republic of Korea; jeongkun@inha.ac.kr

**Keywords:** stem cells, prostate cancer, carboxylesterase

## Abstract

Castration-resistant prostate cancer (CRPC) remains difficult to treat with conventional chemotherapy. We evaluated a stem cell-based enzyme-prodrug strategy using hTERT-immortalized adipose-derived stem cells engineered to express rabbit carboxylesterase 2 (hTERT-ADSC.CE2) in combination with irinotecan (CPT-11). hTERT-ADSC.CE2 cells were generated via lentiviral transduction and confirmed to overexpress CE2. Their tumor-homing capacity toward PC3 prostate cancer cells was assessed, along with prodrug activation, apoptosis induction, and in vivo tumor suppression in a CRPC mouse model. hTERT-ADSC.CE2 cells demonstrated enhanced migration toward PC3 cells and higher expression of tumor-homing factors than the controls. Under CPT-11, they exhibited a strong “suicide” effect and induced selective killing of PC3 cells, with upregulation of BAX and cleaved caspase-3 and downregulation of BCL-2. By day 14, the combination arm showed significantly lower tumor burden than both the control and irinotecan-alone arms (*p* < 0.05). The pattern is consistent with intratumoral activation and localized SN-38 exposure. hTERT-ADSC.CE2 combined with irinotecan provides potent, tumor-targeted cytotoxicity and markedly suppresses CRPC progression. This cell-mediated prodrug activation system may represent a promising therapeutic approach for advanced prostate cancer.

## 1. Introduction

Prostate cancer is one of the most prevalent malignancies in men and the second leading cause of cancer-related mortality worldwide [1,2]. Treatment of advanced disease, particularly castration-resistant prostate cancer (CRPC), remains challenging due to the limited efficacy and high toxicity of conventional chemotherapy [3], highlighting the need for novel therapeutic strategies with improved tumor selectivity and safety.

Gene therapy using tumor-tropic stem cells has emerged as a promising approach for targeted drug delivery [4]. Among these, cell-mediated enzyme/prodrug therapy (EPT) has shown encouraging preclinical results in solid tumors [5]. EPT involves engineering carrier cells to express a prodrug-activating enzyme, which locally converts a non-toxic prodrug into an active chemotherapeutic, leading to high intratumoral drug levels and a bystander effect that eliminates neighboring cancer cells [6,7]. Clinical trials using such strategies, including in malignant gliomas, are ongoing [8].

The cytosine deaminase (CD)/5-fluorocytosine (5-FC) and carboxylesterase (CE)/irinotecan (CPT-11) systems are among the best studied EPT platforms [6]. While CD/5-FC generates 5-fluorouracil (5-FU), which primarily kills proliferating cells [7], this limits efficacy in solid tumors with low-proliferation regions [9,10]. In contrast, irinotecan requires activation by CE to SN-38, a potent topoisomerase I inhibitor that can kill tumor cells independent of the cell cycle phase [11,12]. Rabbit CE2 is the most efficient CPT-11 hydrolase known, with markedly higher catalytic activity than its human counterparts [13,14], making it an attractive candidate to enhance intratumoral SN-38 generation and therapeutic effect [15,16].

Adipose-derived stem cells (ADSCs) are particularly suitable as gene-delivery vehicles due to their abundance, ease of isolation, tumor-homing properties, and potential for autologous application, thereby avoiding ethical and immunological limitations of neural stem cells [17,18,19]. Immortalization with telomerase reverse transcriptase (hTERT) extends their lifespan without malignant transformation [20,21], enabling the development of standardized and reproducible cell products for therapy [19,22].

In this study, we engineered hTERT-immortalized ADSCs to overexpress rabbit CE2 (hTERT-ADSC.CE2) and tested their ability to target PC3, a CRPC tumor, activate irinotecan, and induce tumor cell death in vitro and in vivo. This CE2/CPT-11 system was also compared with the conventional CD/5-FC platform to evaluate its relative therapeutic potential and translational applicability [23,24].

## 2. Materials and Methods

### 2.1. Cell Culture

The hTERT-immortalized human adipose-derived mesenchymal stem cell line ASC52-Telo (ATCC SCRC-4000™, hereafter referred to as hTERT-ADSC) was obtained from ATCC (Manassas, VA, USA). These cells were grown in Dulbecco’s Modified Eagle Medium (DMEM; Gibco BRL, Grand Island, NY, USA) supplemented with 2 mM L-glutamine, 100 U/mL penicillin, 100 µg/mL streptomycin, and 10% heat-inactivated fetal bovine serum (FBS; Gibco BRL). PC3 human prostate cancer cells (Korean Cell Line Bank, Seoul, Republic of Korea) were maintained under identical culture conditions. All cells were incubated at 37 °C in a humidified 5% CO_2_ atmosphere and passaged with trypsin–EDTA once they reached approximately 80% confluence. All reagents in 2.1 are from Thermo Fisher Scientific, Waltham, MA, USA.

### 2.2. Generation of hTERT-ADSC.CE2 Cell Line

To establish a CE2-overexpressing ADSC model, hTERT-ADSCs were transduced with a recombinant lentivirus carrying the rabbit liver CE2 gene (Gene ID: 100357373). The coding sequence was inserted into a CLV-UbiqC lentiviral vector (ubiquitin promoter-driven, containing puromycin resistance and GFP). Lentiviral particles were generated by calcium phosphate co-transfection of the construct with packaging plasmids into HEK293T cells. Viral supernatants were collected 16–20 h after media replacement. hTERT-ADSCs were exposed to a viral supernatant containing 8 µg/mL Polybrene (Sigma, St. Louis, MO, USA) for 4–6 h at 37 °C, followed by fresh medium replacement. After 48 h, puromycin (Sigma-Aldrich, St. Louis, MO, USA) (3 µg/mL) was applied for 2 weeks to select resistant colonies, which were then expanded into stable hTERT-ADSC.CE2 lines. Successful integration and expression of CE2 were validated via molecular assays.

### 2.3. Molecular Characterization

RT-PCR and Real-Time PCR: Total RNA was isolated from parental and engineered AD-SCs using TRIzol (Invitrogen, Waltham, MA, USA). cDNA synthesis was performed from 1 µg RNA with random primers. CE2 transcripts were detected using specific primers (Forward: 5′-CCATTGGGATGAAAGGAAGA-3′; Reverse: 5′-AGAAAAGGAGGGAGCAGAGG-3′). Genes encoding chemoattractants (VEGF, SCF, SDF-1) and receptors (VEGFR1/2/3, c-Kit, CXCR4) were also amplified (primer sequences in Table 1). GAPDH served as an internal control. Conventional PCR products were visualized by agarose electrophoresis, while qPCR employed SYBR Green (Thermo Fisher Scientific, Waltham, MA, USA) chemistry with ΔΔCt analysis, using triplicates from at least three independent experiments.

Western blot whole-cell lysates were prepared in RIPA buffer (Thermo Fisher, Waltham, MA, USA) with protease inhibitors. Equal protein (20–30 µg) was separated via SDS-PAGE and transferred onto PVDF membranes (Millipore, Burlington, MA, USA). After blocking, the membranes (Merck KGaA, Darmstadt, Germany) were probed with antibodies against Nanog, Oct4, Sox2, BAX, cleaved Caspase-3, BCL-2, and β-actin (Santa Cruz Bio-technology; 1:3000). HRP-conjugated secondary antibodies were applied, and signals were detected by ECL (Thermo Fisher Scientific, Waltham, MA, USA) (Amersham). Densitometry was performed using ImageJ (1.51).

Flow Cytometry: Surface markers were assessed on both parental and CE2-modified AD-SCs. The cells were incubated with antibodies targeting CD29, CD90, CD105 (MSC markers), and CD34, CD45 (hematopoietic markers). Isotype controls were included. The analysis was performedhermo Fisher Scientific, 8 cytometer (Sysmex Partec GmbH, Görlitz, Germany) (Sysmex Partec), with data processed using FCS Express software (v 7.20).

### 2.4. In Vitro Migration Assay

A transwell invasion assay (8 µm pores, Merck Millipore, Burlington, MA, USA) coated with Matrigel was used. The lower wells contained either serum-free medium, WPMY-1 stromal cells (non-tumor control), or PC3 cancer cells. After 24 h of chemoattractant conditioning, hTERT-ADSC or hTERT-ADSC.CE2 cells (1 × 10^5^ in serum-free medium) were seeded in the upper chambers. After 48 h, the migrated cells on the lower membrane surface were fixed, stained (Diff-Quick), and counted in five random fields. Each condition was tested in triplicate.

### 2.5. Chemoattractant Ligand and Receptor Analysis

Mesenchymal stem cell tropism is largely driven by gradients of tumor-secreted chemoattractants [25,26], with the SDF-1/CXCR4, SCF/c-Kit, and VEGF/VEGFR pathways playing central roles [27,28]. To evaluate these mechanisms, qPCR was used to compare the expression of SCF, SDF-1, and VEGF ligands in both parental and CE2-modified ADSCs. Receptors (c-Kit, CXCR4, VEGFR1/2/3) were analyzed in hTERT-ADSC.CE2. GAPDH was used for normalization.

### 2.6. In Vitro Cytotoxicity and Suicide Assays

(a) CPT-11 Sensitivity: hTERT-ADSC.CE2 cells (5 × 10^3^/well, 96-well plates) were treated with CPT-11 (0.01–5 µM) for 72 h. Cell viability was measured via an MTT assay (Promega), with absorbance at 570 nm (Tecan reader Tecan Group Ltd., Männedorf, Switzerland). Data (mean ± SE, n = 4 per group) were normalized to untreated controls.

(b) Co-Culture Cytotoxicity: PC3 cells (1 × 10^4^/well) were co-cultured with ADSC.CE2 (ratio 1:0.05) for 24 h and then treated with CPT-11 (5 µM) for 72 h. The controls included PC3 alone + CPT-11, PC3 + unmodified ADSC + CPT-11, and untreated conditions. Viability was assessed by MTT.

(c) Apoptosis Induction: PC3 cells co-cultured with ADSC.CE2 ± CPT-11 were analyzed by Western blot for BAX, cleaved Caspase-3, and BCL-2, with β-actin as the loading control. Changes in pro- and anti-apoptotic markers indicated apoptotic induction.

### 2.7. In Vivo Tumor Growth Inhibition

All animal procedures complied with NIH guidelines. Male athymic nude mice (20–25 g, 6–8 weeks; Orient Bio, Seongnam-si, Republic of Korea) were injected subcutaneously with PC3 cells (1 × 10^6^) with standard housing, controlled temperature and humidity, and a 12-h light/dark cycle. Once tumors formed (~1 week), the mice were assigned to three groups (n = 5): untreated control, CPT-11 + hTERT-ADSC (control cells), and CPT-11 + hTERT-ADSC.CE2 (therapy). ADSCs (1 × 10^6^) were administered via intracardiac injection, while CPT-11 (1.7 mg/kg) was given intraperitoneally following a 5-day-on/2-day-off × 2-week cycle [29].

Tumor volumes were calculated as (length × width^2^)/2 at days 0 and 14. At the endpoint, tumors were excised for PCR/fluorescence analysis of CE2/GFP presence and for histology (H&E, caspase-3 staining. Euthanasia was performed based on humane endpoints, including a tumor size exceeding 2000 mm^3^ or significant body weight loss.

### 2.8. Statistical Analysis

Data are presented as mean ± SE unless otherwise stated. Longitudinal tumor volumes (% of baseline) were analyzed using a linear mixed-effects model (REML) with fixed effects for group, time, and their interaction and a random intercept for mouse. Day-14 pairwise contrasts were Sidak-adjusted. Effect sizes are reported as mean differences (Δ, % of baseline) with 95% confidence intervals. For in vitro assays (e.g., cell viability) and other single-time-point endpoints, one- or two-way ANOVA with Tukey post hoc tests was used as appropriate. Randomization was computer-generated; caliper measurements were blinded to group. Prespecified exclusion criteria (e.g., non-tumor-related death, confirmed mis-injection) were applied before unblinding. A two-sided α = 0.05 was used.

## 3. Results

### 3.1. Tumor-Homing Ability of hTERT-ADSC.CE2

**Figure 1:**
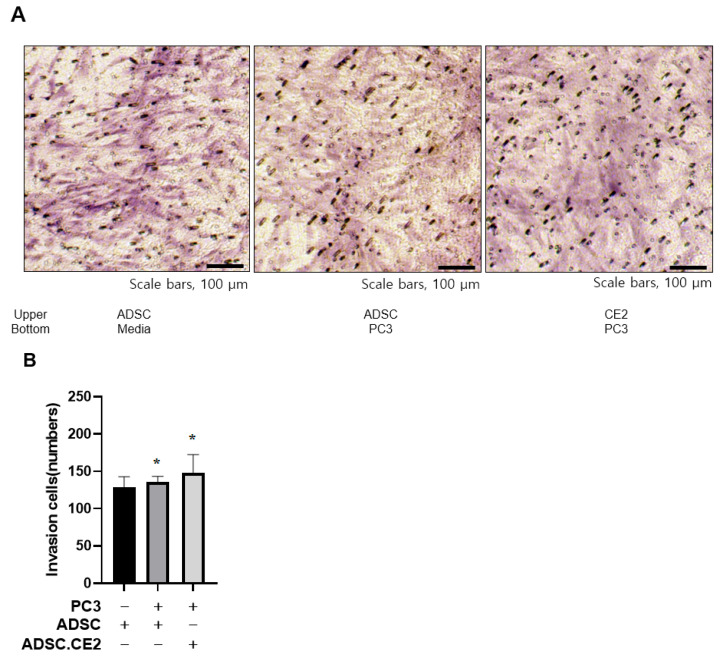
Tumor tropism of CE2-overexpressing hTERT-ADSCs. (**A**,**B**) In vitro invasion assay results: hTERT-ADSC.CE2 cells (and parental hTERT-ADSCs) were tested for migration toward PC3 prostate cancer cells. (**A**): Representative micrographs of stained transwell membranes show numerous ADSC.CE2 cells that migrated to the PC3-containing lower chamber (right), compared to almost none migrating toward the control medium (left). (**B**): Quantification of migrated cells (mean ± SE, n = 3 independent assays). Both hTERT-ADSC and hTERT-ADSC.CE2 exhibited significantly increased migration in the presence of PC3 (*p* < 0.05 vs. medium or WPMY-1 target). There was no significant difference between ADSC and ADSC.CE2, indicating the CE2 transgene did not affect migratory ability. * *p* < 0.05.

**Figure 2:**
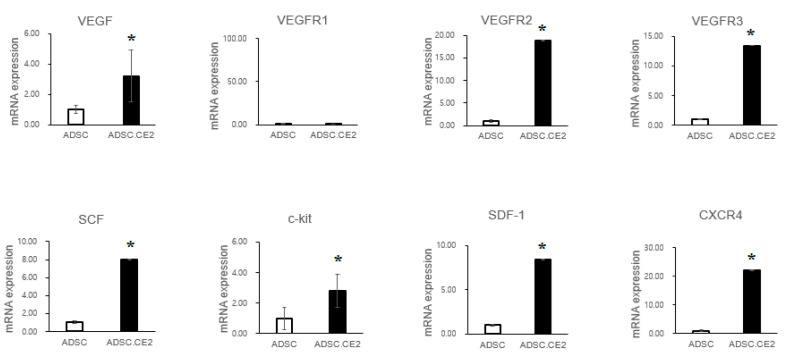
Expression of chemoattractant factors and receptors in hTERT-ADSC.CE2 cells. Real-time PCR analysis comparing unmodified hTERT-ADSC vs. hTERT-ADSC.CE2. The bar graphs show mRNA levels (relative to the ADSC control, set as 1) for key chemoattractant ligands—VEGF, SCF, and SDF-1—and their receptors—VEGFR-2, VEGFR-3, c-Kit, and CXCR4—in ADSC.CE2 cells. The expression of all these factors was higher in hTERT-ADSC.CE2 than in parental ADSCs (* *p* < 0.05 for each). These factors are known to mediate stem cell migration to tumors. Elevated levels in ADSC.CE2 suggest an enhanced readiness to respond to tumor-derived signals. (VEGFR-1 was also tested but showed only baseline expression, not shown.) Error bars represent SE (n = 3). Abbreviations: ADSC = parental hTERT-ADSC; ADSC.CE2 = CE2-transduced hTERT-ADSC. Y-axis: Normalized band intensities relative to β-actin.

Transwell assays showed that hTERT-ADSC.CE2 cells maintained strong tropism toward PC3 cells, comparable to parental ADSCs, but exhibited little migration toward controls (Figure 1A,B). qRT-PCR demonstrated higher expression of chemoattractant receptors (CXCR4, c-Kit, VEGFR2/3) and ligands (VEGF, SCF, SDF-1) in engineered cells compared with unmodified ADSCs (Figure 2). Since PC3 cells also secreted these ligands, the enhanced signaling likely strengthened tumor-homing capacity.

### 3.2. Establishment of CE2-Expressing hTERT-ADSCs

**Figure 3:**
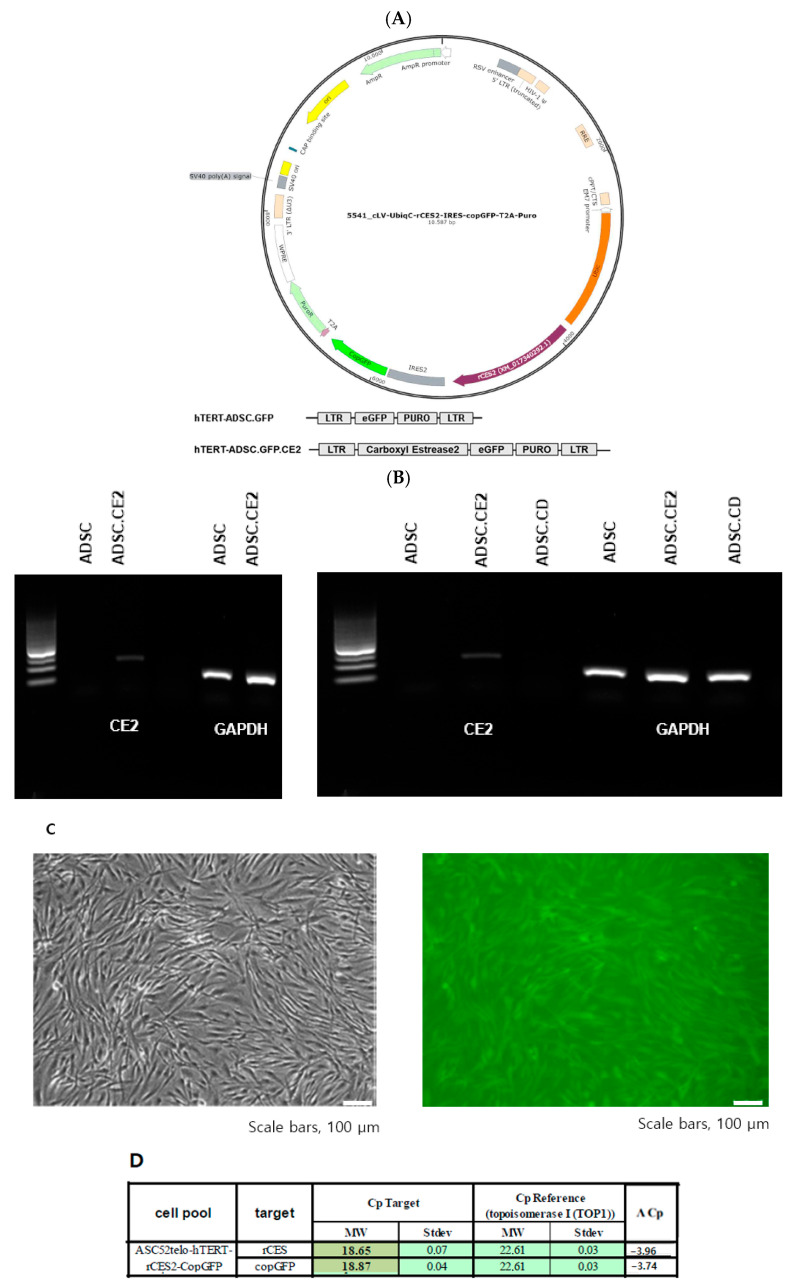
Generation and molecular confirmation of the hTERT-ADSC.CE2 cell line. (**A**): Plasmid maps. hTERT-hADSC and hTERT-hADSC.CE were generated via lentiviral transduction of GFP and the CE gene using the CLV-UbiqC vector. (**B**): RT-PCR showing CE2 gene expression. Lane 1: hTERT-ADSC.CE2 (strong band at expected 200 bp CE2 product); Lane 2: parental hTERT-ADSC (no CE2 band). (**C**): Microscopy of ADSC.CE2 cells. Left: Phase-contrast image showing the typical spindle-shaped morphology of ADSC.CE2 (similar to unmodified ADSCs). Right: Fluorescence image of the same field showing GFP expression in ADSC.CE2 cells (green fluorescence indicates transgene presence). (**D**): Real-time PCR quantification of the CE2 transcript in hTERT-ADSC.CE2 vs. parental cells. The ADSC.CE2 line shows a large increase in CE2 mRNA (ΔCp = −3.96, corresponding to ~16-fold higher expression relative to baseline). No CE2 mRNA was detected in ADSC or ADSC.CD. These data confirm the successful integration and high-level expression of the rabbit CE2 gene in the engineered ADSCs.

Successful creation and molecular profile of the hTERT-ADSC.CE2 line was performed (Figure 3A). Following lentiviral transduction and puromycin selection, nearly all cells expressed GFP (from the bicistronic construct), confirming efficient transduction. RT-PCR revealed robust expression of rabbit CE2 mRNA in hTERT-ADSC.CE2 cells, whereas no signal was detected in parental cells (Figure 3B). Correspondingly, the CE2 protein was absent in the control ADSCs, highlighting the specificity of the modification. Quantitative real-time PCR demonstrated markedly elevated CE2 transcript levels, exceeding baseline expression by more than an order of magnitude (Figure 3D). Fluorescence microscopy also showed abundant GFP-positive cells, with distinct perinuclear fluorescence patterns, confirming successful integration of the construct (Figure 3C).

**Figure 4:**
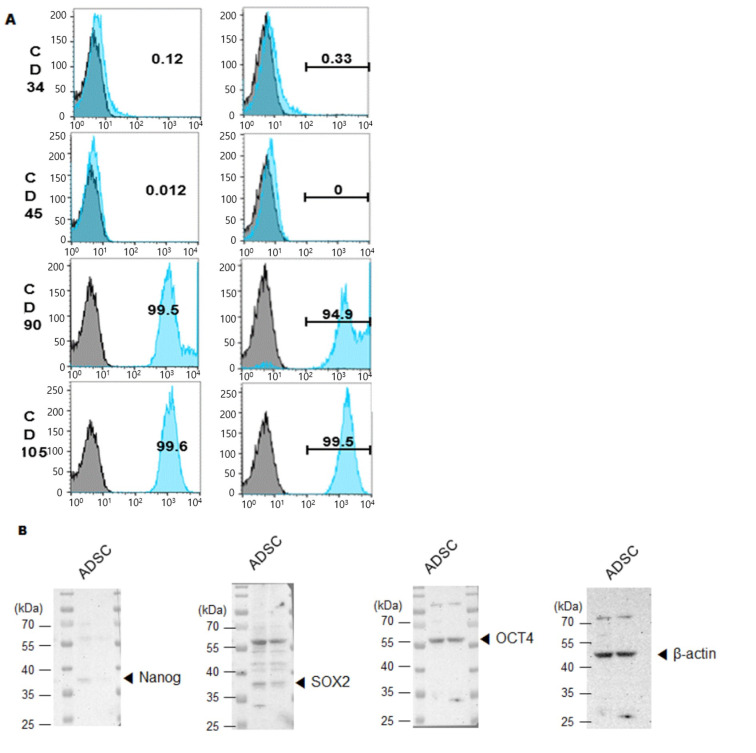

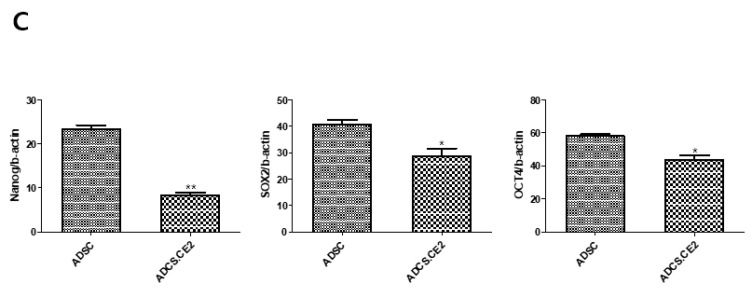
Phenotypic characterization of hTERT-ADSC.CE2 cells. (**A**): Flow cytometry for surface markers. Histograms show that hTERT-ADSC.CE2 cells (shaded curves) are positive for mesenchymal stem cell markers CD29, CD90, and CD105 and negative for hematopoietic markers CD34 and CD45, identical to parental ADSCs. The overlay of ADSC vs. ADSC.CE2 was nearly superimposable for each marker (isotype control indicated by dotted line). Y-axis: Cell count. (**B**): Western blot analysis of stemness markers Oct4, Nanog, and Sox2 in parental and CE2-modified ADSCs. Both cell lines express these transcription factors, indicating maintenance of stem cell characteristics. (**C**): Densitometric quantification of band intensities from (**B**) (normalized to β-actin). There were significant differences in Oct4/Nanog/Sox2 levels between hTERT-ADSC and hTERT-ADSC.CE2, confirming that the introduction of hTERT and CE2 did not diminish the cells’ stemness. Overall, hTERT-ADSC.CE2 retains a typical MSC immunophenotype and pluripotency marker expression profile, validating it as a bona fide mesenchymal stem cell line. Y-axis: Normalized band intensities relative to β-actin. * *p* < 0.05, ** *p* < 0.01.

Phenotypic stability was then evaluated to ensure that CE2 expression did not alter MSC identity. Flow cytometry showed that hTERT-ADSC.CE2 cells strongly expressed MSC surface markers CD29, CD90, and CD105 while lacking hematopoietic markers CD34 and CD45 (Figure 4A). Western blot analysis further confirmed the presence of core stemness markers (Oct4, Sox2) at levels comparable to parental ADSCs. However, Nanog expression is quite weak in hTERT-ADSC.CE2 cells compared to ADSCs (Figure 4B,C). Morphologically, hTERT-ADSC.CE2 retained the fibroblast-like, spindle-shaped appearance of ADSCs under phase-contrast microscopy (Figure 3C, left panel), with no evidence of abnormal differentiation or growth.

### 3.3. Tumor-Selective Cytotoxicity In Vitro

#### 3.3.1. In Vitro Tumor-Selective Cytotoxicity of hTERT-ADSC.CE2 with CPT-11

**Figure 5:**
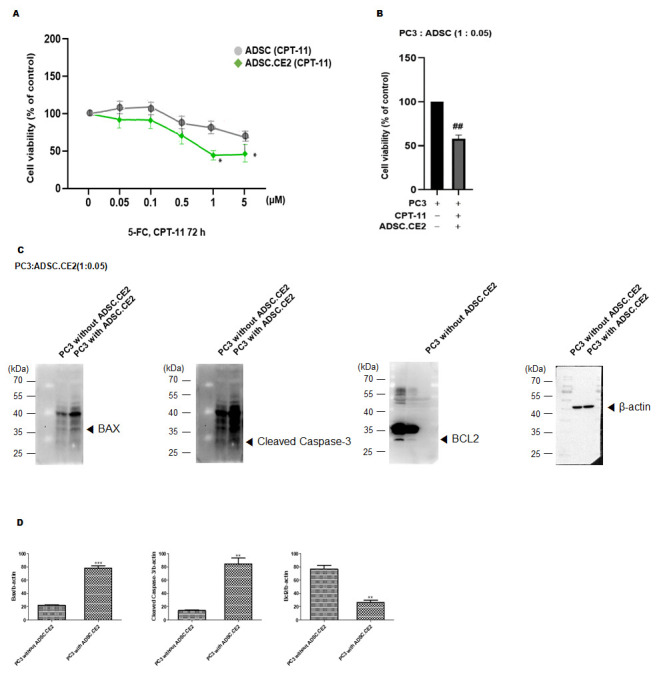
In vitro therapeutic effects of the ADSC.CE2 + CPT-11 system. (**A**): Suicide effect on ADSCs: Viability of hTERT-ADSC.CE2 vs. hTERT-ADSC after 72 h exposure to increasing CPT-11 concentrations. Data are mean % of untreated control viability ± SE (n = 4). At CPT-11 ≥ 1 µM, ADSC.CE2 viability drops significantly below ADSC viability (*p* < 0.05), indicating CE2-mediated conversion of CPT-11 to SN-38 kills the CE2-expressing cells. (**B**): Selective cytotoxicity in co-culture: PC3 prostate cancer cells were co-cultured with either ADSC or ADSC.CE2 (1:0.05 ratio) and treated with CPT-11 (5 µM, 72 h). Cell viability is expressed relative to the untreated PC3 control. Co-culture with ADSC.CE2 led to ~40% viability of PC3, significantly lower than PC3 + CPT-11 alone (~80%) or PC3 + ADSC + CPT-11 (~85%) (*p* < 0.05). This demonstrates a strong bystander killing effect on cancer cells due to SN-38 release by ADSC.CE2. (**C**): Apoptosis induction: Western blot images of co-cultured PC3 cells (after 72 h CPT-11) show increased BAX and cleaved caspase-3 and decreased BCL-2 in the PC3 cells when ADSC.CE2 were present (right lane) compared to control conditions (PC3 + CPT-11 without ADSC.CE2, left lane). (**D**): Densitometry of apoptosis protein levels (each normalized to β-actin, and expressed relative to control = 1). The ADSC.CE2 + CPT-11 co-culture caused ~2-fold increase in BAX and cleaved caspase-3 and ~50% reduction in BCL-2, confirming activation of apoptosis in tumor cells (*p* < 0.05). PC3: PC3 alone, CPT-11: PC3 + CPT-11, ADSC.CE2: PC3 + CPT + 11 + ADSC.CE2. Y-axis: Normalized band intensities relative to β-actin. Together, these results highlight that the enzyme/prodrug therapy not only kills cancer cells but does so by triggering apoptotic pathways. (Error bars = SE, n = 3 experiments.) ** *p* < 0.01, *** *p* < 0.001, ## *p* < 0.01.

As shown in Figure 5A, hTERT-ADSC.CE2 viability declined in a dose-dependent manner following CPT-11 exposure, whereas unmodified ADSCs remained largely unaffected at comparable doses. At ≥1 µM CPT-11, CE2-expressing cells showed significantly reduced viability compared to parental ADSCs (*p* < 0.05). At 5 µM, about 50% of the engineered cells were eliminated within 72 h, while the control ADSCs largely survived (Figure 5A). The co-culture of hTERT-ADSC.CE2 with PC3 cells under 5 µM CPT-11 treatment demonstrated a marked reduction in PC3 viability compared with CPT-11 alone or CPT-11 with control ADSCs (Figure 5B). Even at a low ADSC-to-PC3 ratio of 1:20, CE2-expressing cells significantly enhanced tumor killing (*p* < 0.05). Western blot analysis showed increased expression of pro-apoptotic BAX and cleaved caspase-3, along with reduced anti-apoptotic BCL-2, in PC3 cells treated with CPT-11 in the presence of CE2-expressing ADSCs (Figure 5C). Densitometry confirmed significant changes in BAX/β-actin, cleaved caspase-3/β-actin, and BCL-2/β-actin ratios (Figure 5D).

#### 3.3.2. In Vivo Anti-Tumor Efficacy

**Figure 6:**
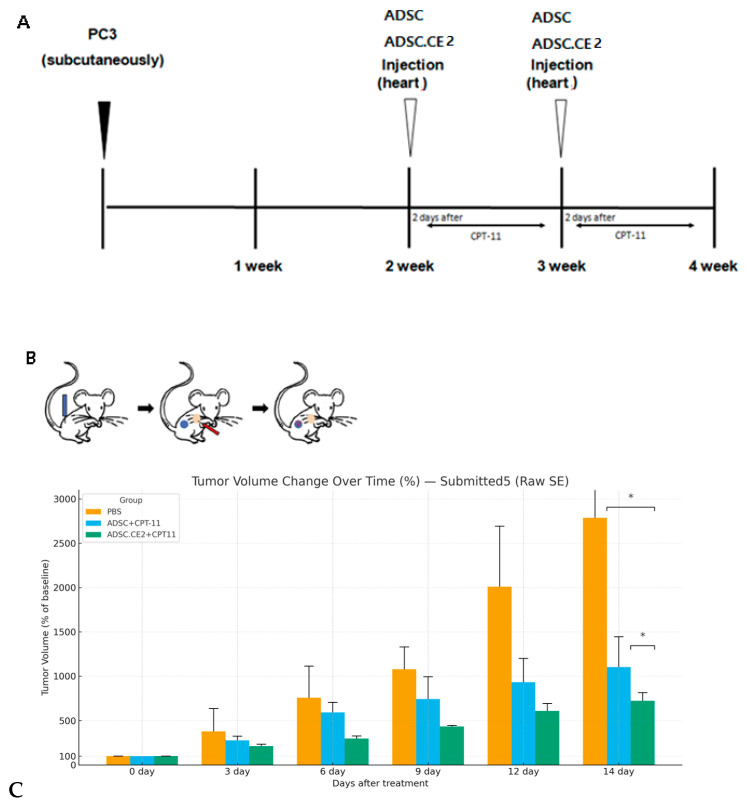
In vivo anti-tumor efficacy of systemic hTERT-ADSC.CE2 and CPT-11 treatment. (**A**): Schematic timeline of the treatment protocol. PC3 tumors were established on day 0. On day 7, mice received intracardiac injection of PBS (control), hTERT-ADSC (for the CPT-11 alone group), or hTERT-ADSC.CE2 (for the combination group). CPT-11 was administered intraperitoneally in two cycles (5 days on, 2 days off, 5 days on) during weeks 1 and 2. Tumor volumes were measured on days 0 and 14. (**B**): Illustration of the therapeutic mechanism. PC3 tumor (blue) is implanted; hTERT-ADSC.CE2 cells (red) are injected and home to the tumor; the hTERT-ADSC.CE2 cells (red) become intermixed with the PC3 tumor (blue) through homing; subsequent CPT-11 injections result in localized SN-38 production at the tumor site by the CE2-expressing cells, killing tumor cells. (**C**): Tumor growth results. The graph shows the tumor volume (mean ± SE, n = 5 per group) on day 14 as a percentage of the initial volume. Y-axis: Tumor volume at each time point expressed as a percentage of the initial tumor volume prior to treatment. Untreated tumors grew to ~2790% of the starting size. CPT-11 alone slowed growth (~1100%), while the ADSC.CE2 + CPT-11 group had only ~720% of the initial volume. The combination therapy achieved significantly greater tumor growth inhibition than CPT-11 alone or the control (*p* < 0.05 for both comparisons). Tumor volume over time as % of baseline (Day 0 = 100%) for PBS, ADSC+CPT-11, and ADSC.CE2+CPT11. Error bars indicate SEM. On day 14, the combination group differs significantly from PBS and ADSC+CPT-11 (asterisks). * *p* < 0.05.

Nude mice bearing subcutaneous PC3 tumors were treated with no therapy, CPT-11 alone, or the combination regimen. Tumor growth was most strongly suppressed in the ADSC.CE2 + CPT-11 group compared with both the untreated and CPT-11 alone groups (Figure 6C). By day 14, tumor volumes were ~721 ± 92% of the baseline in the combination group, compared with ~2786 ± 994% in the controls (*p* < 0.05) and ~1103 ± 414% in the CPT-11 group (*p* < 0.05 vs. control and vs. combination). In vivo, systemic delivery of hTERT-ADSC.CE2 with CPT-11 significantly suppressed tumor growth, reducing the tumor volume to ~26% (721%/2786%) of the control tumor volume. Figure 6A outlines the treatment schedule, while Figure 6B illustrates the therapeutic strategy: systemically delivered hTERT-ADSC.CE2 cells home to tumors and locally convert CPT-11 to SN-38.

## 4. Discussion

Cell-mediated gene therapy represents a promising strategy for targeted cancer treatment, with EPT being a central component [30]. By using cells—particularly stem cells—to deliver prodrug-activating enzymes directly to tumors, EPT maximizes local drug concentrations while minimizing systemic toxicity. In this study, we applied an EPT system using hTERT-immortalized ADSCs expressing rabbit CE2, combined with CPT-11, to treat PC3, the CRPC model, a stage where conventional chemotherapies show limited efficacy [1,3]. Our findings indicate that this system effectively targets prostate cancer cells and achieves strong anti-tumor effects in vitro and in vivo.

Previous work with enzyme/prodrug pairs, such as CD/5-FC, has demonstrated tumor suppression in prostate cancer and other models [19,20,31]. However, 5-FU primarily kills rapidly dividing cells, leaving quiescent tumor populations unaffected and potentially causing systemic toxicity at high doses [7,12,32]. In contrast, the CE2/CPT-11 system produces SN-38, a potent topoisomerase inhibitor capable of killing cells across multiple cell cycle phases [11,31], with better tissue penetration and an extended bystander effect [15]. Systemic CPT-11 conversion to SN-38 in humans is inefficient [33,34,35], but local CE2 expression in ADSCs significantly enhances intratumoral SN-38 production, as confirmed by the in vitro suicide effect observed at ≥1 µM CPT-11 (Figure 5A) [13,14].

Prior studies using CE/CPT-11 EPT with neural stem cells (NSCs) demonstrated tumor inhibition in prostate and pancreatic cancer models with minimal toxicity [22,36]. However, NSCs pose ethical, sourcing, and immunogenicity challenges, whereas ADSCs are easily obtained, expandable, and can be used autologously [13,17,18,37,38]. Our use of hTERT-immortalized ADSCs (ASC52-Telo) ensures a consistent, unlimited cell supply while retaining MSC markers, morphology, and multipotency after CE2 transduction (Figure 2), consistent with prior reports on hTERT-immortalized MSCs [19,20,22].

A key safety feature is the self-limiting nature of the therapy: CE2-expressing ADSCs undergo apoptosis upon CPT-11 treatment, providing a built-in suicide mechanism that eliminates carrier cells after prodrug activation (Figure 5A, in vivo observations) [38,39]. This reduces the risk of unwanted tissue formation or tumor support by MSCs. Notwithstanding these observations, the use of hTERT-immortalized ADSCs and a xenogeneic (rabbit) CE2 entails potential risks—including carrier-cell tumorigenicity/genomic instability and CE2 immunogenicity—that were not evaluated here. Dedicated safety studies (e.g., an ADSC-only control, karyotyping/anchorage-independent growth, in vivo persistence/biodistribution, and cytokine profiling) are therefore warranted in follow-up work [25]. A major limitation of our study is that it is confined to the PC3 cell line, which does not account for the biological diversity of CRPC.

In our CRPC model, systemic administration of hTERT-ADSC.CE2 combined with CPT-11 markedly inhibited tumor growth compared with CPT-11 alone or the untreated controls (Figure 6C). Minimal toxicity was observed in normal tissues, consistent with local SN-38 generation at tumor sites. The data confirm robust tumor tropism, selective cancer cell killing, and effective in vivo anti-tumor activity, highlighting the advantages of combining stem cell homing with the potent irinotecan/SN-38 system.

While enzyme/prodrug efficacy varies with tumor type, prodrug pharmacokinetics, bystander range, and carrier tropism, our findings indicate that CE2/CPT-11 delivered via ADSCs outperforms earlier CD/5-FC strategies in CRPC [15]. The use of patient-derived ADSCs further supports potential clinical translation.

This feasibility study did not include pharmacokinetic/pharmacodynamic analyses—specifically LC–MS/MS quantification of SN-38, CPT-11, and APC in tumor versus plasma/major organs; assessments of CE2 expression and enzymatic activity in tumor and non-tumor tissues; and biodistribution/persistence of hTERT-ADSC.CE2 after systemic delivery. Accordingly, the mechanistic interpretation remains inferential and will be examined in subsequent studies.

In summary, genetically engineered hTERT-ADSC.CE2 cells provide a targeted, self-limiting delivery platform for local activation of CPT-11 (SN-38) within the tumor microenvironment. This approach showed tumor-selective cytotoxicity and growth inhibition in a PC3 xenograft model and supports further evaluation of ADSC-mediated enzyme/prodrug therapy in advanced prostate cancer.

## 5. Conclusions

hTERT-immortalized human ADSCs overexpressing carboxylesterase 2, combined with irinotecan, exhibited potent and tumor-selective anti-cancer effects against PC3, a castration-resistant prostate cancer model. These engineered ADSCs efficiently migrated toward prostate tumors and localized in vivo, enabling on-site conversion of CPT-11 into its active metabolite SN-38. This resulted in strong, selective cytotoxicity and apoptosis in cancer cells while simultaneously eliminating the therapeutic ADSCs, reducing the risk of long-term engraftment. The therapy significantly inhibited tumor growth in both in vitro co-culture and in vivo mouse models, outperforming irinotecan alone. Compared with the conventional CD/5-FC suicide gene system, the CE2/CPT-11 approach offers enhanced efficacy and practical advantages, including the use of easily obtained autologous ADSCs and a clinically established prodrug. These results underscore the potential of CE2-expressing ADSCs as a precise, safe, and effective cell-based therapy for PC3 prostate cancer, supporting its future evaluation in clinical trials.

## Figures and Tables

**Table 1:** PCR primer sequences for key genes (Forward and Reverse sequences are shown 5′→3′, with expected PCR product size in base pairs).

Gene	Forward Primer Sequence	Reverse Primer Sequence	Product Size (bp)
CE2	5′-CCATTGGGATGAAAGGAAGA-3′	5′-AGAAAAGGAGGGAGCAGAGG-3′	200
SCF	5′-ACTTGGATTCTCACTTGCATTT-3′	5′-CTTTCTCAGGACTTAATGTTGAAG-3′	505
c-Kit	5′-GCCCACAATAGATTGGTATTT-3′	5′-AGCATCTTTACAGCGACAGTC-3′	332
SDF-1	5′-ATGAACGCCAAGGTCGTGGTC-3′	5′-GGCTGTTGTGCTTACTTGTTT-3′	200
CXCR4	5′-CTCTCCAAAGGAAAGCGAGGTGGACAT-3′	5′-AGACTGTACACTGTAGGTGCTGAAATCA-3′	733
VEGF	5′-AAGCCATCCTGTGTGCCCCTGATG-3′	5′-GCTCCTTCCTCCTGCCCGGCTCAC-3′	541
VEGFR1	5′-GCAAGGTGTGACTTTTGTTC-3′	5′-AGGATTTCTTCCCCTGTGTA-3′	512
VEGFR2	5′-ACGCTGACATGTACGGTCTAT-3′	5′-GCCAAGCTTGTACCATGTGCG-3′	438
VEGFR3	5′-AGCCATTCATCAACAAGCCT-3′	5′-GGCAACAGCTGGATGTCATA-3′	298

## Data Availability

The data presented in this study are available on request from the corresponding author due to ethical reasons.
